# Supplementary material for: Antithrombin Activity and Association with Risk of Thrombosis and Mortality in Patients with Cancer
Source: Int J Mol Sci. 2022 Dec 12;23(24):15770. doi: 10.3390/ijms232415770 (PMC9784494; doi:10.3390/ijms232415770)
Supplement: Supplementary file 1 [file ijms-23-15770-s001.zip › ijms-1995701-supplementary.pdf]

Supplementary Materials

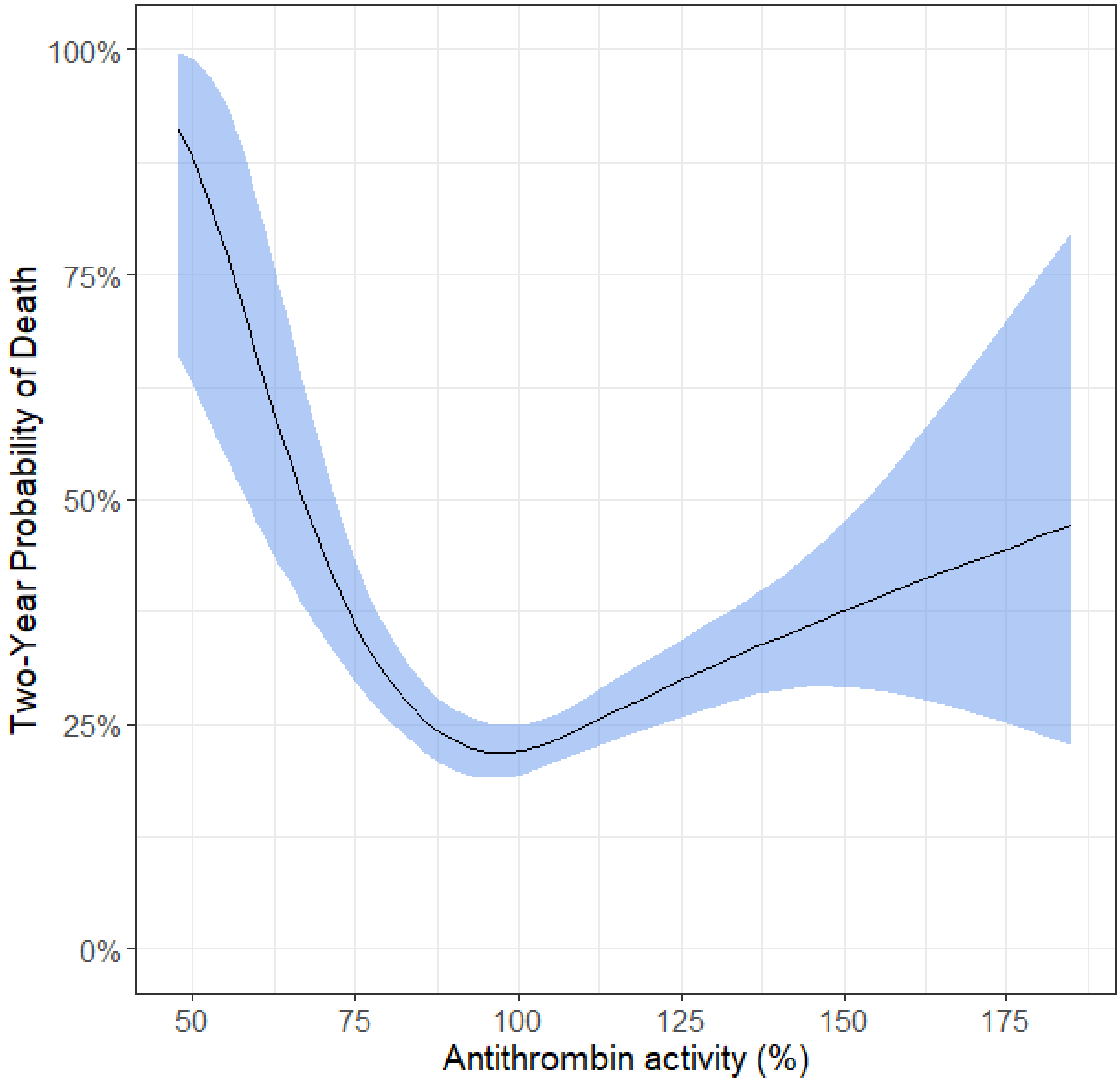

Supplementary Figure S1. Probability of death after 1 year of follow-up depending on antithrombin activity level. Y axis depicts the one-year probability of death in %, i.e. 100% meaning a 100% probability of death at 1 year follow-up. Blue shaded area indicates 95% Confidence Interval.

Supplementary Table S1. Events (VTE/ATE) in patients that died during the follow-up period (n=563)

Event	n	
	Yes	No
**VTE,** n (%)	65 (11.5%)	498 (88.5%)
**ATE,** n (%)	21 (3.7%)	542 (96.3%)

Supplementary Table S2. Antithrombin activity levels according to different cancer types.

Antithrombin activity levels						
**Cancer type**	**n**	**Minimum**	**25th percentile**	**Median**	**75th percentile**	**Maximum**
Brain+	194	48	109	120	131.25	185
Breast	156	67	98	108	115	139
Lung	253	50	91,5	101	111	149
Stomach	33	68	90	98	108.5	131
Colorectal	113	54	91.5	98	108	134
Pancreatic	104	51	88	98.5	108.75	167
Kidney	27	75	94	102	108	118
Prostate	107	73	92	100	108	125
Multiple Myeloma	7	67	89	97	101	106
Lymphoma	45	71	92	100	108.5	121
Others*	98	68	95	104.5	11.25	134

*Esophageal, sarcoma, testis, hepatocellular, thymus, genitourinary, thyroidal, mesothelioma

+Including only primary brain tumors: glioma, medulloblastoma, meningioma

Supplementary Table S3. Correlation of Antithrombin activity levels and acute-phase proteins.

	**r**	**95% Confidence Intervall**	**p**
Antithrombin and D-dimer	-0.119	(-0.179) – (-0.058)	<0.001
Antithrombin and CRP	-0.346	(-0.429) – (-0.257)	<0.001
Antithrombin and Fibrinogen	-0.113	(-0.172) – (-0.053)	<0.001

Supplementary Table S4. Hazard ratios for all-cause mortality with varying antithrombin activity levels. Ref - Reference

	**Hazard ratio (95% Confidence Interval)**
**1st quartile**	1.49 (1.14-1.95)
**2nd quartile**	Ref.
**3rd quartile**	0.99 (0.75-1.31)
**4th quartile**	1.52 (1.18-1.97)
**1st quartile**	1.51 (1.16-1.97)
**2nd quartile**	1.01 (0.77-1.33)
**3rd quartile**	Ref.
**4th quartile**	1.54 (1.19-1.98)
**1st quartile**	1.51 (1.2-1.89)
**2nd quartile**	Ref.
**3rd quartile**	Ref.
**4th quartile**	1.53 (1.23-1.89)
**1st/4th vs 2nd/3rd quartile**	1.52 (1.26-1.82)

Supplementary Table S5. Hazard ratios for all-cause mortality in brain tumor patients with varying antithrombin activity levels. Ref - Reference

	**Hazard ratio (95% Confidence Interval)**
**1st quartile**	Ref.
**2nd quartile**	1.85 (1.02-3.36)
**3rd quartile**	1.66 (0.91-3.02)
**4th quartile**	2.76 (1.56-4.88)
**≤120 % Antithrombin activity level**	Ref.
**>120% Antithrombin activity level**	1.59 (1.07-2.36)
